# Authors’ response: Measles outbreak in Gothenburg urban area, Sweden, 2017/18: lower viral load in breakthrough infections

**DOI:** 10.2807/1560-7917.ES.2019.24.30.1900478

**Published:** 2019-07-25

**Authors:** Nicklas Sundell, Leif Dotevall, Magnus Lindh, Johan Westin, Jan-Åke Liljeqvist, Tomas Bergström, Marie Studahl, Lars-Magnus Andersson

**Affiliations:** 1 Department of Infectious Diseases, Institute of Biomedicine, Sahlgrenska Academy, University of Gothenburg, Gothenburg, Sweden; 2 Department of Communicable Disease Control, Region Västra Götaland, Sweden

**Keywords:** measles, outbreak, PCR, vaccine, immunity, serology

**To the editor:** We appreciate the interest in our report. We agree, as stated in our paper, that there are reports of onward transmission of measles from cases with breakthrough infections in household settings. However, we do not conclude that the risk of measles transmission is limited to unvaccinated cases, but rather that ”*…there was a large difference in viral load in nasopharyngeal samples between patients with naïve and breakthrough infections of measles, and our results indicate that a high risk of onward transmission is confined to naïve infections*” [1]. Thus, with our definition of breakthrough infection (history of vaccination and detectable IgG (high avidity) at rash onset (or within 4 days)), the risk of onward transmission is low in most cases. We agree that Ct values are likely to be of great value, but we were not able to define any cut-off value above which onward transmission is unlikely. As shown in our report, the overlap regarding Ct values in nasopharyngeal samples is less pronounced using our definition than in the report by Seto et al. [2]. In fact, 14 of our 16 patients with breakthrough infections had Ct values above 30 (or were negative by PCR) in nasopharyngeal samples. It is possible that contact tracing for these 14 individuals could have been restricted to close contacts, such as household members. The reports by De Serres et al. and Eom et al. are referred to as examples of onward transmission from vaccinated individuals [3,4]. However, no data regarding IgG levels and avidity at rash onset (or within 4 days) are presented in the reports and it is not possible to know if these cases represent true breakthrough infections. In the report by Avramovich et al., IgG levels and avidity are presented, but the index case was identified retrospectively (at least 14 days after rash onset) and it is not stated when the serum sample was taken [5]. It is thus not possible to evaluate whether this presumed index case actually had pre-existing immunity or not.

In summary, we firmly believe that our definition of breakthrough infection of measles is clinically useful and can be used to guide contact tracing in areas with high vaccination coverage. Semi-quantitative determination of measles viral load in nasopharyngeal samples or throat swabs at rash onset are probably of value to evaluate the risk of onward transmission, but cut-off values remain to be determined. We encourage other groups to repeat our observations in new outbreak settings in areas with high vaccination coverage.
